# A novel perineal exposure method in laparoscopic abdominal perineal resection of rectal cancer: a case series study

**DOI:** 10.1186/s12893-024-02453-9

**Published:** 2024-05-20

**Authors:** Jun Ma, Daibin Tang, Yuquan Tang, Datian Wang, Peng Jiang, Yaming Zhang

**Affiliations:** 1 Department of General Surgery, Anqing Municipal Hospital, No. 352, Ren-Ming Road, Anqing, 246000 Anhui Province P.R. China; 2 Department of Anesthesiology, Anqing Municipal Hospital, Anqing, 246000 P.R. China

**Keywords:** Rectal cancer, Abdominal perineal resection, Lone-star® retractor, Surgery, Method

## Abstract

**Background:**

Abdominal perineal resection (APR) of rectal cancer, also known as Mile’s procedure, is a classic procedure for the treatment of rectal cancer. Through the improvement of surgical skills and neoadjuvant therapy, the sphincter-preserving rate in rectal cancer patients has improved, even in patients with ultralow rectal cancer who underwent APR in the past. However, APR cannot be completely replaced by low anterior resection (LAR) in reality. APR still has its indications, when the tumor affects the external sphincter, etc. Good perineal exposure in APR is difficult and can seriously affect surgical safety and the long-term prognosis.

**Methods:**

We reviewed the records of 16 consecutive patients with rectal cancer who underwent APR at Anqing Municipal Hospital from January 2022 to April 2023, including 11 males and 5 females, with an average age of 64.8 ± 10.3 years. The perineal operation was completed with the Lone-Star® retractor-assisted (LSRA) exposure method. After incising the skin and subcutaneous tissue, a Lone-Star® retractor was placed, and the incision was retracted in surrounding directions with 8 small retractors, which facilitated the freeing of deep tissues. We dynamically adjusted the retractor according to the plane to fully expose the surgical field.

**Results:**

All 16 patients underwent laparoscopic-assisted APR successfully. Thirteen procedures were performed independently by a single person, and the others were completed by two persons due to intraoperative arterial hemostasis. All specimens were free of perforation and had a negative circumferential resection margin (CRM). Postoperative complications occurred in 4 patients, including urinary retention in 1 patient, pulmonary infection in 1 patient, intestinal adhesion in 1 patient and peristomal dermatitis in 1 patient, and were graded as ClavienDindo grade 3 or lower and cured. No distant metastasis or local recurrence was found for any of the patients in the postoperative follow-up.

**Conclusions:**

The application of the LSRA exposure method might be helpful for perineal exposure during APR for rectal cancer, which could improve intraoperative safety and surgical efficiency, achieve one-person operation, and increase the comfort of operators.

## Introduction

Colorectal cancer is the fourth most lethal cancer in the world, and approximately 900 thousand people die from colorectal cancer each year [1]. The incidence of rectal cancer is steadily increasing every year, and 70–80% of rectal cancer lesions involve the middle and lower rectum [2, 3]. Moreover, the rate of rectal cancer increased in younger people [4]. Abdominal perineal resection (APR) of rectal cancer, also known as Miles procedure, is a classic procedure for the treatment of rectal cancer[5]. Currently, almost all Miles procedures are performed laparoscopically, and the dominance of open surgery has been superseded [6, 7].

Through the improvement of surgical skills and neoadjvant therapy, the sphincter-preserving rate in rectal cancer patients has improved, even in patients with ultralow rectal cancer who underwent APR in the past [8–12]. However, APR cannot be completely replaced by low anterior resection (LAR) in reality [13]. APR might be a better choice for patients with low rectal cancer with invasion of the external anal sphincter or levator anus, an inability to achieve safe margins when performing LAR.

The success of APR relies on good perineal exposure. First, patients must be placed in the lithotomy position, with full abduction of both hips to expose the operative field and to better accommodate two surgeons for perineal operations. However, some elderly men or patients with hip disease were often unable to achieve adequate abduction of both legs. Second, in some medical institutions, surgeons sometimes changed the patients’ position to the prone folded knife position during perineal operations to achieve better exposure of the operative field. However, the authors believed that this operation was time-consuming and increased the risk of adverse events. Finally, to expose the operative field, the assistant often needed to suspend the arms, which not only required physical strength but also lacked the continuity of traction.

The Lone-Star® retractor (Cooper Surgical Inc., Connecticut, USA) is beneficial because it is simple to use, reliable and allows good exposure, and is currently reported to be used in pediatric surgery, hemorrhoids, transanal total mesorectal excision (TaTME), intersphincteric resection (ISR), natural orifice specimen extraction surgery (NOSE), stoma reduction, and neurosurgery [14–20]. However, the Lone-Star retractor-assisted (LSRA) exposure method has not been reported in APR. In this study, we will describe the application of this method in APR.

## Materials and methods

### Patients

We reviewed the records of 16 consecutive patients with rectal cancer who underwent APR at Anqing Municipal Hospital from January 2022 to May 2023, in which the perineum was exposed using the Lone-Star® retractor. The inclusion criteria were patients who were diagnosed with low rectal cancer by pathology and imaging (the lower edge of the lesion was less than 6 cm from the anal verge). In addition, one of the following conditions was met at least: (1) late local staging and refusal of neoadjuvant therapy; (2) no guaranteed safe margin when performing low anterior resection (LAR); and (3) lesion encroaching on the external anal sphincter or the levator anus muscle.

All procedures were performed in accordance with the ethical standards of the responsible committee on human experimentation (institutional and national) and with the Declaration of Helsinki 1964 and later versions. The present study was approved by the Medical Ethics Committee of Anqing Municipal Hospital (ref. no. 2023. 058). All patients or their representatives provided informed consent for inclusion in the study.

All patients underwent preoperative routine examinations, including colonoscopy, MRI, and computed tomography, for staging, diagnosis and tumor localization. The diagnosis of rectal cancer was confirmed by pathological examination. It was confirmed intraoperatively that there were no distant metastases, such as hepatic or, or peritoneal metastases.

### Surgical procedure

**Fig. 1:**
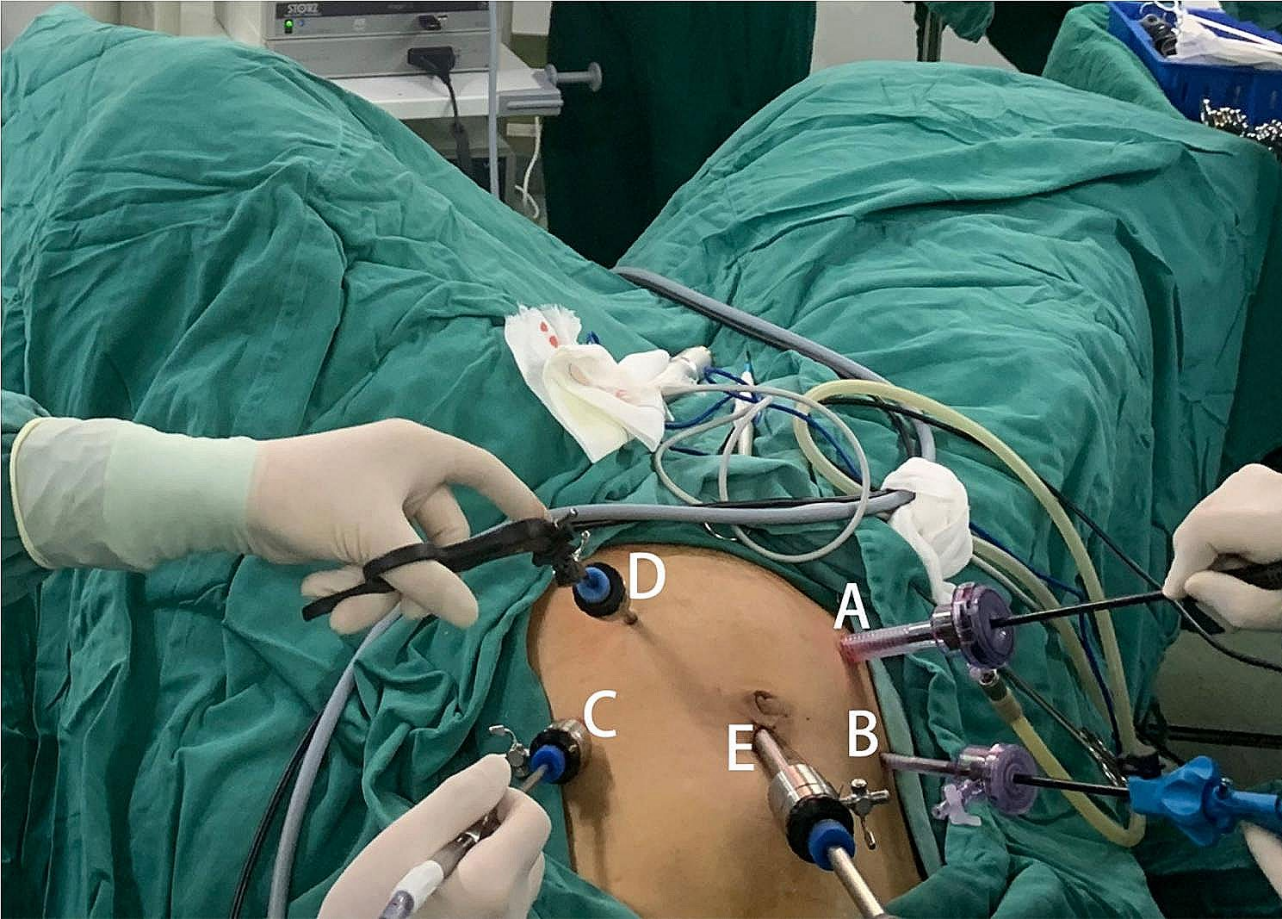
Layout of the trocars **A**: A 12 mm trocar was placed two transverse fingers inside the right anterior superior iliac spine as the main operation hole for the surgeon. **B**: A 5 mm trocar was placed at the right midclavicular line at the flat umbilicus as the auxiliary operation hole for the surgeon. **C**: A 5 mm trocar was placed at the left midclavicular line at the flat umbilicus as the main operation hole for the first assistant. **D**: A 5 mm trocar was placed at the left Mai’s point as an auxiliary operation hole for the first assistant. **E**: A 10 mm diameter trocar was placed 1 cm above the umbilicus as an observation hole

After completion general anesthesia, the patient was placed in the lithotomy position, and the conventional five-hole method was performed (Fig. 1). The abdominal operation needed three operators (a surgeon was positioned on the right side of the patient, a first assistant on the left side, and a second assistant on the cephalad side).

### Abdominal procedure

The operator performed laparoscopic detachment of the left Toldt’s space, ligated and sectioned the vessel at the root of the inferior mesenteric artery (IMA), and ligated and sectioned the inferior mesenteric vein (IMV) distally from the confluence of the left colonic vein.

We sectioned the sigmoid mesentery along the marginal vascular arch down to 15 cm proximal to the tumor. Then, the rectum was separated posteriorly along the posterior rectal space to the tip of the coccyx according to the TME principle.

The peritoneum was incised anteriorly in an arc 1 cm above the peritoneal reflex line, and the peritoneum was separated to the lower margin of the seminal vesicle glands in males and to the lower margin of the uterine cervix in females.

The lateral rectum needed to be dissected to the inferior border of the pelvic autonomic nerves bilaterally. Finally, the rectum would be dissected to the plane of the levators and the sigmoid colon was excised with a 60 mm linear stapler (one firing) 10 cm above the tumor.

After the above steps were completed, the surgeon moved to the perineum to perform the perineal operation(LSRA exposure method), and the other two assistants continued to complete the colostomy and closure of the abdominal trocar incision.

### Perineal operation(LSRA)

We closed the anus using the purse-string suture method and made a circular incision along the anus, which ranged from the tip of the coccyx at the posterior border to the perineal center point anteriorly and 2 cm from the anal verge on the lateral side.

**Fig. 2:**
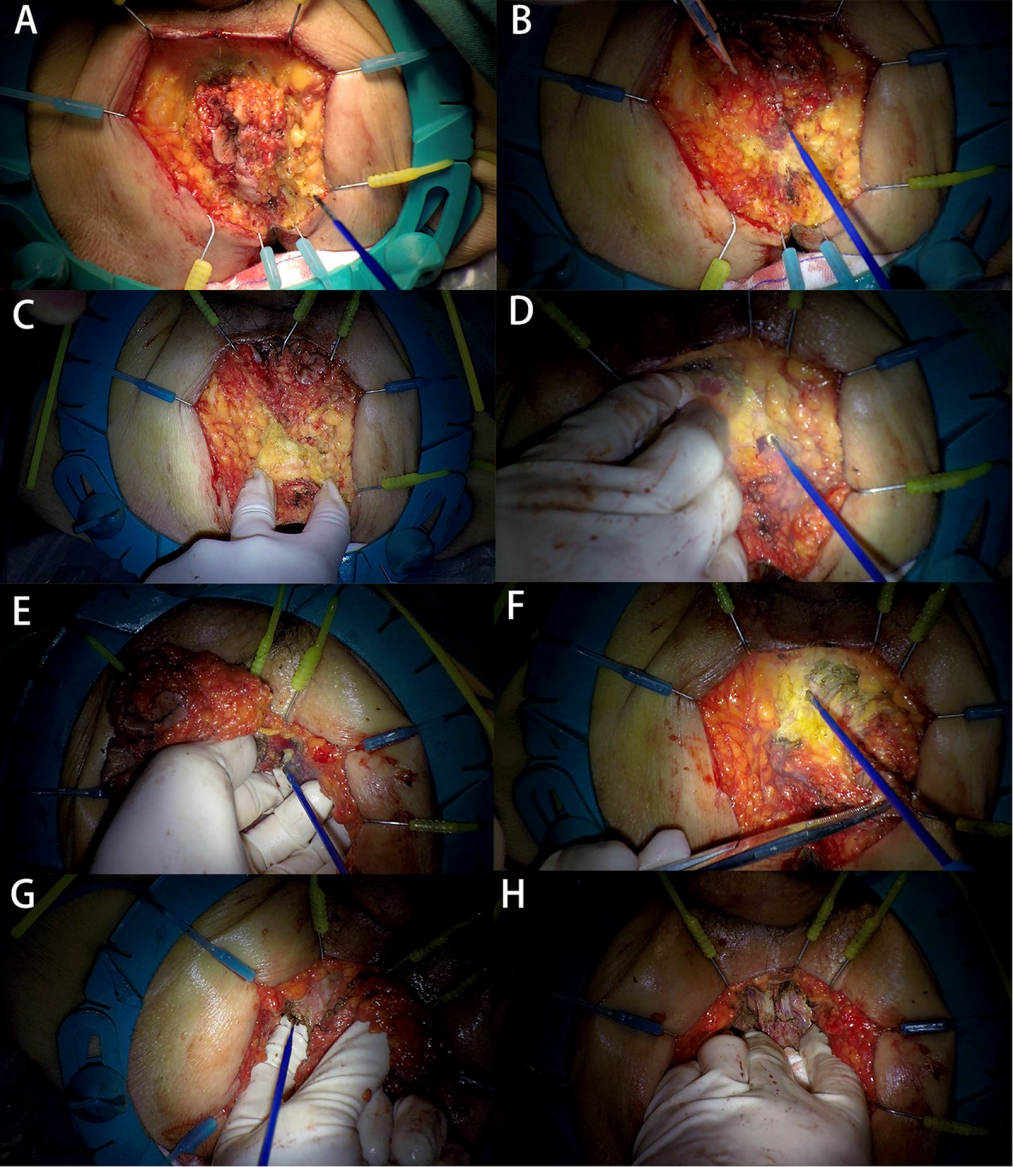
Application of the LSRA method for perineal exposure **A**: After incision of the skin and subcutaneous tissue, a Lone-star® retractor was placed. **B-C**: We used the anterior 3 small hooks to pull the anal canal tissue toward 12 o’clock. **D-E**: The left levator anus muscle was dissected. **F-G**: The right levator anus muscle was dissected. **H**: The anterior rectal wall tissue needed to be detached

After incising the skin and subcutaneous tissue, a Lone-Star retractor® was placed, and the incision was retracted in surrounding directions with 8 small hooks, which facilitated the freeing of deep tissues (Fig. 2A).

When posterior freeing to the caudal anal ligament, we used the anterior 3 small hooks to pull the anal canal tissue toward 12 o’clock, and the surgeon could symmetrically pull the posterior fat tissue in the opposite direction, which could fully expose the posterior surgical area (Fig. 2B-C).

After opening the caudal anal ligament to communicate with the pelvis, the operator’s left fingers reach into the pelvis, utilizing the index and middle fingers as an anatomical guide, and then proceed to extend the incision of the levator anus muscle.

The authors were accustomed to the order of dissecting the left levator anus muscle first and then the right side. The reason was that when the left levator anus muscle was dissected, the thumb of the left hand was able to push the rectal tissue to the right side to better perform the procedure (Fig. 2D-E).

After the posterior and left tissues had been freed, the surgeon then proceeded to incise the right levator anus muscle (Fig. 2F-G). It should be noted that the vessels in the 1 and 11 o’clock directions needed to be preprocessed, thus preventing intraoperative arterial bleeding.

**Fig. 3:**
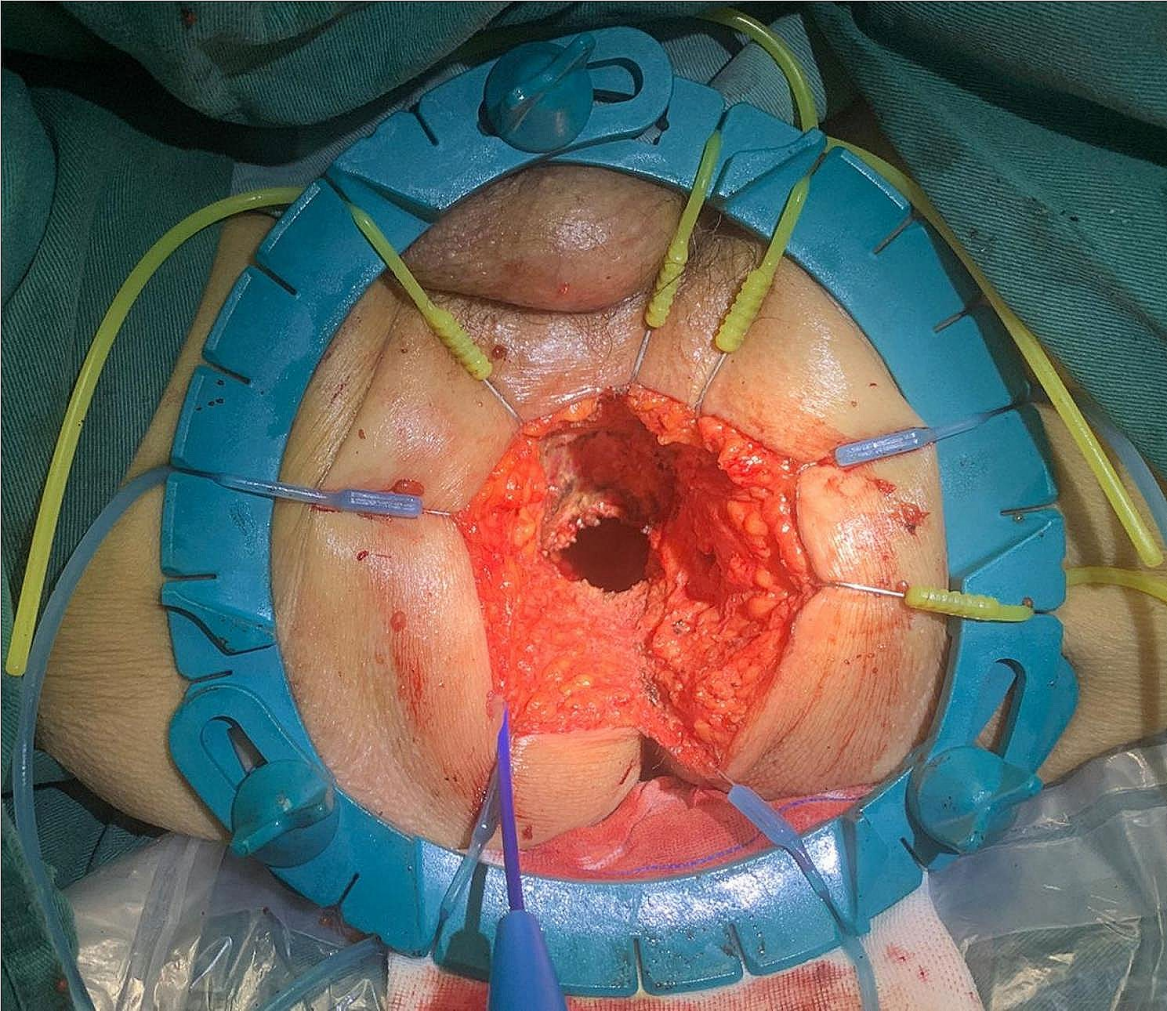
Display of perineum after removal of specimen

Finally, only the anterior rectal wall tissue needed to be detached (Fig. 2H). The surgeon placed the remaining attached tissue between the index finger and thumb of the left hand and subsequently pulled the specimen toward the 6 o’clock position and held tension to complete the anterior dissection. After removal of the specimen(Fig. 3), we flushed the wound, placed one drainage tube in the presacral area, and closed the perineal incision.

## Results

### Patient characteristics

A total of 16 patients underwent laparoscopic APR without combined organ resection, including 11 males and 5 females, with a mean age of 64.8 ± 10.3 years and BMI of 22.8 ± 3.8 kg/m^2^. The tumor distance from the anal verge was 3.2 ± 1.3 cm, and the tumor size was 4.1 ± 1.0 cm. Two patients underwent preoperative conversion therapy, 1 patient had significant tumor progression, and 1 patient had poor defecation control function. None of the patients had ascites, peritoneal metastasis or liver metastasis in intraoperative exploration. (Table 1).

**Table 1:** Clinicopathological characteristics

Variables		*N* = 16
Age (year)		64.8 ± 10.3
Sex		
Male Female		11 5
Body mass index(kg/m2)		22.8 ± 3.8
Distance of tumor from anal verge(cm)		3.2 ± 1.3
Tumor size(cm)		4.1 ± 1.0
Preoperative treatment		
None Chemoradiotherapy		14 2
Pathological stage		
I II III IV		6 5 5 0
Surgical approach		
Open Laparoscopy		0 16

Values are presented as the mean ± standard deviation or median (range)

### Surgical results

All 16 patients were successfully treated with laparoscopic-assisted APR, in which 13 perineal operations were performed by a single person independently, and the others were completed by two persons due to intraoperative arterial hemostasis. The operative time was 260 (100–310) min, the perineal operative time was 53.8 ± 15.0 min, and the intraoperative bleeding was 100 (50–200) ml. All specimens were free of perforation and had a negative circumferential resection margin (CRM). The postoperative ventilation time was 3.5 ± 1.6 days, the presacral drainage tube was removed on postoperative Day 6 (3–14), and the postoperative length of stay was 8.5 (6–18) days. (Table 2)

**Table 2:** Surgical outcomes

Variables		*N* = 16
Postoperative first bowel movement(days)		3.5 ± 1.6
Postoperative hospital stay (days)		8.5(6–18)
Operation time (min)		260(100–310)
Perineal operation time (min)		53.8 ± 15.0
The time of withdraw drainage tube(days)		6(3–14)
Perforation of specimen		
Yes None		0 16
CRM		
Positive Negative		0 16
Number of people needed for perineal operations		
Single Two		13 3
Blood loss (ml)		100(50–200)

Values are presented as the mean ± standard deviation or median (range)

### Postoperative complications and follow-up outcomes

Postoperative complications occurred in 4 patients, accounting for 25% of all patients. Urinary retention occurred in 1 patient, pulmonary infection in 1 patient, intestinal adhesion in 1 patient and peristomal dermatitis in 1 patient. All complications were graded as ClavienDindo grade 3 or lower and cured (Table 3). All patients underwent blood tests and imaging examinations every 3–6 months, the last follow-up time was January 2024, and no metastasis or recurrence was found to date in the postoperative follow-up.

**Table 3:** Clavien‒Dindo classification of postoperative complications

Complication	No. of cases	Clavien‒Dindo classification						
		I	II	IIIa	IIIb	IVa	IVb	V
Pneumonia	1		1					
Intestinal obstruction	1		1					
Urinary retention	1		1					
Peristomal dermatitis	1	1						
Total	4	1	3	0	0	0	0	0

## Discussion

The self-retaining retractor has been applied in surgery since 1964, when it was used to expose the abdominal cavity because of its exceptional continuity, cost-effectiveness, and ability to facilitate good exposure [21]. Owing to its improved technology, the Lone-Star retractor has been used in a variety of surgical procedures. Through our clinical practice, the LSRA method played an important role in perineal exposure in APR procedures.

Advantages of the LSRA method for perineal exposure in rectal cancer resection. (1) The retractor can be freely adjusted, allowing the operative area to be fully visualized during the perineal operation, which is especially suitable for difficult pelvic surgeries or pelvic surgeries involving males as it facilitates the intraoperative identification of vital organs, thereby reducing the incidence of surgical complications and the rate of perforation of the anterior wall. (2) Application of the LSRA exposure method allows two assistants to perform colostomy after completing the abdominal operation while the surgeon moves to the perineum to perform the perineal operation without the need for additional surgical staff. Otherwise, it was necessary to wait for the completion of the abdominal operation before proceeding to the perineal operation; avoiding a wait time between procedures increases the efficiency of the operation and reduces the staffing requirements. (3) Some patients’ right thigh could not be excessively abducted during laparoscopic-assisted APR, thus possibly interfering with the operator’s maneuverability in IMA lymph node dissection. Our maneuver technique makes perineal manipulation easier in this group of patients. (4) There was no need to change positions intraoperatively by applying the LSRA exposure method, which eliminated the possible surgical and cardiovascular risks associated with changing positions and greatly reduced operative time. (5) With the help of the LSRA exposure method, it was possible to perform perineal operation with one surgeon.

There are several advantages of the single-person operation. First, the operating space was limited, and the assistant’s hands were suspended for a long time in the previous perineal surgery, which caused severe pain in the arms and shoulders after the operation. On the other hand, some patients could not fully abduct their legs, and only one person could be in the surgical area of the perineum, which was a better way to show the function of the lone-star retractor. In our cases, 13 procedures were completed by one surgeon, and only 3 procedures were completed by two operators because of initial inexperience of the surgeons and intraoperative bleeding. We concluded that intraoperative preligation of the lateral anal artery could be an important step in preventing active hemorrhage and could contribute to the completion of a single-person operation.

The pathologic margins were negative in all 16 cases, no severe postoperative complications occurred, and there were no reoperation cases. Urinary retention occurred in a 70-year-old male and was cured after conservative treatment. The incidence of urinary retention in our cases was 6.3%. According to the literature, the incidence of postoperative urinary retention in rectal cancer patient ranges from 5.4 to 52% [22,23], and the incidence might be higher in the APR procedure, which is associated with postoperative pain, low tumor location, nerve injury, male sex and advanced age.

There are some key points to keep in mind when applying the LSRA exposure method during perineal surgery. First, because of the sharp head end of the small hooks, it was important for the operators to prevent accidental injury when removing or fixing the lone-star retractor . Second, it was important to keep the head end of the small hooks from touching the tumor tissue to prevent implantation of the incision, although the incidence of this was very rare [24]. Finally, we tried to avoid pulling blood vessels, and if necessary, gauze could be placed over the area to protect it from intraoperative side injuries.

Finally, studies with larger sample sizes and regular follow-up are needed, and the related variables need to be observed, including postoperative pain, sexual function, and long-term prognosis. Considering the limitations of the single-arm study in our article, we will follow up with a controlled study to further validate the clinical value of our novel exposure technique in the future.

## Conclusion

In summary, the LSRA exposure method may be valuable for perineal exposure during APR for rectal cancer, which could improve intraoperative safety and surgical efficiency, achieve one-person operation, and increase the comfort of operators.

## Data Availability

Data is provided within the manuscript.

## Abbreviations

APR: Abdominal perineal resection
LAR: Low anterior resection
LSRA: Lone-Star® retractor-assisted
CRM: Circumferential resection margin
TaTME: Transanal total mesorectal excision
ISR: Intersphincteric resection
NOSE: Natural orifice specimen extraction surgery
IMA: Inferior mesenteric artery
IMV: Inferior mesenteric vein
